# Transport Vehicles as a Vector of Goose Parvovirus Infections (GPV)

**DOI:** 10.3390/ani15243572

**Published:** 2025-12-11

**Authors:** Wojciech Kozdruń, Paweł Olszowiec, Karolina Piekarska, Jowita S. Niczyporuk

**Affiliations:** 1 Department of Virology and Virological Animal Diseases, National Veterinary Research Institute, Partyzantów 57, 24-100 Puławy, Poland; wkozdrun@piwet.pulawy.pl (W.K.); selket23@wp.pl (K.P.); jowita.niczyporuk@piwet.pulawy.pl (J.S.N.); 2 Faculty of Transport, Electrical Engineering and Computer Science, Casimir Pulaski Radom University, 26-600 Radom, Poland

**Keywords:** goose parvovirus, goose, transport vehicles, Derzsy’s disease, GPV

## Abstract

One of the greatest epizootic threats to waterfowl is infection with goose parvovirus (GPV), which is the etiological agent of Derzsy’s disease (DD). Despite the use of prophylactic vaccinations, the disease still occurs in waterfowl. The aim of this study was to conduct a preliminary assessment of the presence of GPV genetic material in transport vehicles based on the results of molecular testing of samples collected from the vehicles between 2017 and 2020. In selected disinfected transport vehicles transporting geese from hatcheries to farms and from farms to slaughterhouses, swabs were collected from transport cages and transport kennel equipment. Samples were collected between 2017 and 2020. Total DNA was then isolated, and PCR amplification was performed to detect the presence of GPV genetic material. The resulting PCR products were sequenced. Based on PCR results, the presence of genetic material from GPV strains originating from disinfected waterfowl transport vehicles was confirmed. From the collected materials, 13 strain sequences were obtained from 2017 to 2020. Based on molecular studies conducted between 2017 and 2020, it should be concluded that despite preventive vaccinations and disinfection of transport vehicles, GPV infection remains a serious epizootic problem in waterfowl flocks in Poland.

## Simple Summary

One of the causes of infections in geese, despite preventive vaccinations, may be traces of the virus in insufficiently or inadequately disinfected transport vehicles that take geese from hatcheries to farms and from farms to slaughterhouses. Therefore, we assessed the risk of these infections, considering that despite the existence of means to disinfect transport, GPV infection remains a serious epizootic problem.

## 1. Introduction

Poland, together with Hungary, is one of the largest producers of geese in the European Union and the third largest in the world, just behind China and Egypt [1]. The intensification of poultry production and the constantly growing number of goose flocks in Poland increase the risk of infectious diseases. Geese produced in Poland are mainly intended for consumption purposes [2,3]. The most frequently reported viral disease in fattening geese in Poland is Derzsy’s disease caused by goose parvovirus (GPV), and this disease is included under the registration rule [4].

Waterfowl parvoviruses constitute a serious epizootic and economic problem in large-scale poultry production. GPV belongs to the Dependovirus genus and Parvoviridae family, causing Derzsy’s disease in Muscovy ducklings (*Cairina moschata*) and goslings (*Anser anser domestica*) with high morbidity and mortality [5]. Its genome is composed of single-stranded DNA (ssDNA), typically spanning around 5000 to 5200 nucleotides in length. The gene-coding segment of this virus is divided into three structural proteins (VP1, VP2, and VP3) and two non-structural proteins (NS1 and NS2). The three structural proteins are critical in the assembly of the viral capsid and are instrumental in the viral capability to infect host cells; the two non-structural proteins are crucial for the initial phases of GPV replication [6]. The 58 kDa molecular weight VP3 protein is the main capsid protein of the virus, the main protective antigen, and contains the main epitope of the virus. This protein can induce the body to produce neutralizing antibodies and provide protective cross-immunity to other waterfowl parvoviruses; results of investigations showed that the VP3 protein is the main candidate antigen for vaccine development and serodiagnostic tests [7,8,9,10,11,12]. The progression of Derzsy’s disease may be acute, subacute, or chronic. Acutely infected geese show clinical signs including anorexia, nasal and ocular discharge, conjunctivitis, severe diarrhea, hepatitis, hydropericardium, myocarditis, and, sometimes, mucosal necrosis in the oral cavity. Furthermore, one- to three-week-old geese may develop a prolonged form of disease upon recovery from the initial infection. Typical pathological conditions associated with the disease include pulmonary edema, pericarditis, perihepatitis, hepatic/myocardial lesions, and ascitic fluid accumulation in the abdomen, and exhibit fibrinous and necrotic enteritis, as well as small intestinal embolism, which is formed when the surface of the intestinal mucosa dies and falls off [1,5,13]. The GPV spreads both vertically through infected eggs and horizontally: directly through infected feces, and indirectly through contaminated equipment, litter, and even through people moving between farms [4,14].

Transport of waterfowl can play a significant role in the spread of parvovirus, particularly through the movement of infected birds or contaminated materials. This can lead to outbreaks in previously unaffected areas or flocks. The virus is highly contagious and can be shed in feces and potentially contaminate surfaces like transport vehicles. If infected birds are transported, their feces could leave the virus on the vehicle, which could then infect other birds when the vehicle is used again or if it comes into contact with other waterfowl. Transport vehicles, especially if not properly cleaned and disinfected after carrying infected birds, can become a source of indirect transmission.

Due to the fact that goose parvovirus is excreted in feces in large quantities, it is characterized by high resistance to environmental conditions and high survival in the environment; therefore, studies have been undertaken to initially assess the presence of GPV genetic material in transport vehicles as potential vectors of GPV infections.

## 2. Materials and Methods

**Preparation of samples for examination:** Samples were collected from selected waterfowl transport vehicles in the form of swabs from transport cages and fixed equipment within the transport vehicles. Samples were collected between 2017 and 2020. The obtained swabs were suspended in Eagle’s solution (MEM, Sigma Aldrich, St. Louis, MO, USA) with 1% antibiotics mixture (Antibiotic-Antimycotic, Gibco, Paisley, UK). The swabs were then removed, and the solution was subjected to a triple freeze–thaw cycle to recover virus particles. The prepared material was centrifuged for 5 min at 4 °C at 3000× *g*, and the supernatant was collected for total DNA isolation. Genetic material was isolated using a commercial kit (Indispin Pathogen Kit, Indical, Leipzig, Germany) according to the manufacturer’s instructions. Extracted DNA templates were stored below −20 °C for PCR analysis.

**Reference DNA.** Goose parvovirus H strain isolated from the commercial Palmivax vaccine was used as a positive control to assess the presence of GPV genetic material. The cellular DNA isolated from uninfected goose embryo fibroblast (GEFs—goose embryo fibroblast) cells was used as a negative control.

**Primers for PCR.** The primer sequences were complementary to the conserved regions of the genome of the virus examined: the region of the genome encoding the GPV VP3 protein: VP3F-5′ GTG CCG ATG GAG TGG GTA AT 3′; ′VP3R-5 GCG CCA GGA AGT GCT TTA T 3′. The sequence of these primers was prepared in the PRIMER computer program and synthesized by a commercial company, GENOMED, Warsaw. The primers were prepared for testing in accordance with the manufacturer’s protocol.

**PCR analysis**. The PCR reaction was performed in a 25 μL reaction mixture containing: 2.5 μL PCR buffer (EurX, Gdańsk, Poland); 1 μL of a mixture of deoxynucleotides (0.2 mM); 1 μL of each primer (10 mM); 3 μL of isolated DNA; 0.5 μL of Taq DNA polymerase (1 μL); 1 μL MgCl_2_ (25 mM); 4 μL betaine solution (Betaine, Sigma Aldrich, USA) and 11 μL of PCR water. The thermal conditions for the reaction of amplification were as follows: 40 cycles; initial denaturation 95 °C/5 min; denaturation—94 °C/15 s; annealing primers—60–20 s; chain extension—72 °C/45 s; and final chain extension—72–10 min.

**Electrophoresis**. PCR products were separated on a 2% agarose gel with GelRed™ Nucleic Acid Gel Stain (Biotium, Fremont, CA, USA). MassRuler Ladder LR (80–1031 bp) was used as a fragment size standard. Electrophoretic separation was performed at 120 V for 50 min in TEA buffer (50XAppliChem Pancreac, AppliChem GmbH, Darmstadt, Germany). Separation results were read under UV light (Biorad, Hercules, CA, USA). Agarose gels were photographed and documented using a gel documentation system (VWR Genosmart, Darmstadt, Germany). The PCR results were considered positive if the DNA product with the expected size of 1604 bp was visible under UV light at a wavelength of 302 nm.

**Sequencing and phylogenetic analysis.** Positive PCR products were sequenced using the Sanger method (Genomed, Warszawa, Poland). Multiple alignments were constructed using the ClustalW method of the software MEGA (Version 7). After alignment, abnormal sequences, too long/short sequences, and sequences with low similarity were removed. Phylogenetic analyses were carried out in the MEGA version 7.0 program using the neighbor-joining method (N-J) and the bootstrap validation method with 1000 replications. Homologous strains used for comparisons and further phylogenetic analysis were searched in the GenBank database, National Center for Biotechnology Information (NCBI) using the BLAST 2.17.0 (Basic Local Alignment Search Tool).

**Goose embryo fibroblast (GEF) cell culture.** Goose embryo fibroblast cell cultures were prepared from 14-day-old goose embryos according to generally accepted principles. The goose embryos came from breeding flocks of geese that were free of goose parvovirus (GPV) and other infectious diseases (Indykpol, Lublin, Poland). The growth medium was Eagle’s fluid medium (MEM, Sigma Aldrich, USA) enriched with 10% fetal bovine serum (Gibco, UK) and 1% antibiotic mixture (Antibiotic-Antimycotic, Gibco, UK). The resulting cell density was approximately 1 × 10^6^ in 1 mL of suspension.

**Propagation of GPV strains in goose embryo fibroblast (GEF) cultures**. Immediately after pouring the resulting GEF cell suspension into sterile adherent culture bottles (Nunc™, Thermo Fisher Scientific, Roskilde, Denmark), 1 mL of the initial viral material was inoculated with a dilution of 10^−1^ per 10 mL of goose embryo fibroblast cell culture. This material was filtered through sterile filters (Minisart Syringe Filter, Sartorius, Göttingen, Germany) with a pore diameter of 0.45 μm. The material for infecting the GEF cultures consisted of 10 supernatants from homogenates obtained from swabs from transport vehicles containing GPV genetic material, which had previously been confirmed by PCR testing. Infected GEF cultures were incubated for approximately 120 h at 37.5 °C in a 5% CO_2_ atmosphere with 85% humidity (Heraeus, Hera Cell, Hanau, Germany). GEF cultures were observed daily under a light microscope, assessing and documenting the resulting cytopathic effect (CPE). The observed changes in the uniform cell culture layer indicated virus multiplication and the appearance of a cytopathic effect. After 5–7 days, the GEF culture flasks were refrigerated and then frozen and thawed three times. Four subsequent passages of each viral material were performed according to the methodology described above, and viral materials from the fourth passage constituted a pool for further studies. The prepared viral material was preserved after each passage and stored at a temperature below −20 °C until further work.

**Determination of infectious titer (TCID_50_) in goose embryo fibroblast (GEF) cell cultures**. The infectious titer of the standard H strain isolated from the commercial Palmivax vaccine and field strains of goose parvovirus was determined in 24-well Nunclon plates (Nunc™, Denmark) covered with a uniform layer of GEF cells. To prepare the culture for titration, 1 mL of a GEF cell suspension at a density of approximately 1 × 10^6^ cells/mL was added to each well on the plate. The plates were incubated for 24 h at 37.5 °C, 85% relative humidity, and an atmosphere supplemented with 5% CO_2_. The growth medium was then discarded, and the GEF cell culture was washed three times with Eagle’s solution and infected with viral material in previously prepared dilutions (10^−1^–10^−7^) in a volume of 0.1 mL. The infected culture was assessed daily under a light microscope, observing and documenting the appearance of cytopathic effect (CPE). After 72 h of incubation, the TCID_50_ titer (50% tissue-culture infectious dose) was calculated based on the presence of CPE according to the Reed–Muench formula [15].

## 3. Results

**Figure 1:**
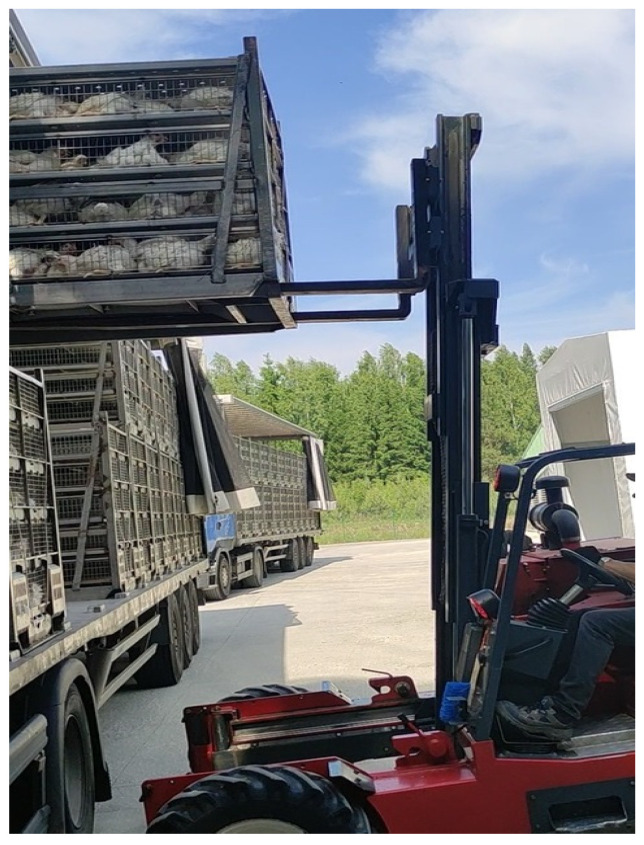
Example of loading birds in transport cages into a transport vehicle (W. Kozdruń).

Poultry transport can be a significant route for viral infections because vehicles used to transport live birds, as well as the birds themselves, can carry and spread viruses, contaminating the environment. Vehicles used to transport poultry can become contaminated with feces, feathers, and other virus-carrying materials. Contaminated vehicles can then spread these viruses to other farms or locations, even if they do not directly transport live birds (Figure 1).

Samples for analysis. In 2017, swabs were collected from 15 waterfowl transport vehicles from various locations. GPV genetic material was detected in six of these vehicles, and three GPV sequences were used for phylogenetic analysis. In 2018, swabs were collected from 22 waterfowl transport vehicles from various locations. GPV genetic material was detected in nine of these vehicles, and three GPV sequences were used for phylogenetic analysis. Swabs were collected from 17 transport vehicles in 2019. Goose parvovirus genetic material was detected in five of these vehicles, and four GPV sequences were used for phylogenetic analysis. In 2020, swabs from 12 transport vehicles were examined, with positive results obtained in five of these vehicles, and three goose parvovirus sequences were used for further analysis. This summary is presented in Table 1.

**Figure 2:**
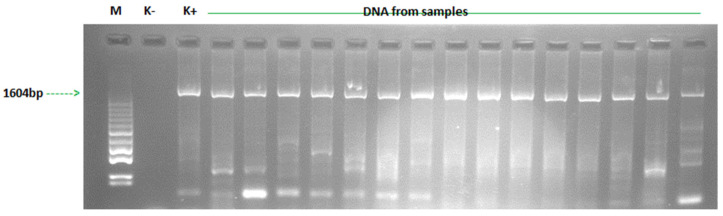
The result of PCR for detection of DNA GPV in samples from swabs of examined vehicles. (M)—molecular mass marker (80–10,000 bp); (K−)—negative control, total DNA isolated from uninfected goose embryo fibroblast (GEF) cell cultures, (K+) positive control, total goose parvovirus DNA isolated from a commercial vaccine against Derzsy’s disease; (DNA from samples) amplicons obtained with VP3-F and VP3-R primers specific for VP3 protein.

**Amplification of the VP3 gene of GPV strains originating from transport vehicles**. Total DNA was isolated from swabs collected from transport cages and vehicles using a commercial kit. This isolated DNA served as a template for PCR amplification using primers complementary to the genomic sequence encoding the GPV capsid VP3 protein. Of the 25 GPV strains identified in waterfowl transport vehicles, sequences from 13 GPV strains were used for phylogenetic analysis of the VP3 capsid protein gene. The selection was based on the quality of the sequences obtained. The sequences of Polish strains were compared with those available in the GenBank (NCBI) database. An example electropherogram showing products of 1604 bp, characteristic of the entire VP3 protein, is shown in Figure 2.

**Figure 3:**
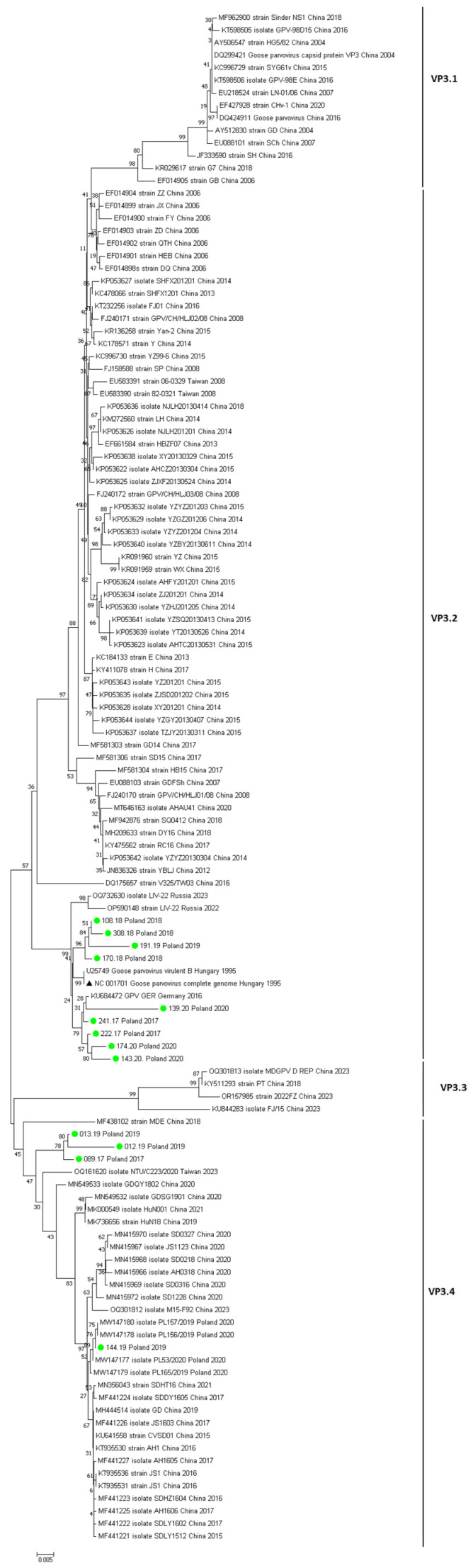
A phylogenetic tree developed based on the nucleotide sequence of the VP3 capsid protein gene of Polish and foreign GPV strain sequences and the sequence of the reference strain (marked with a black triangle). The examined strains are marked with a green circle. Summary diagram (VP3.1–VP3.4).

**Nucleotide and Amino Acid Sequence Analysis.** To characterize the GPV strains identified in transport vehicles in Poland, PCR product sequencing for the conserved capsid protein of the VP3 gene was performed. Products with the best visual quality were selected for sequencing, producing distinct reaction products in the form of bands on a 2% agarose gel under UV light. The primers used allowed for sequencing a 1604 bp fragment. Due to errors occurring during the sequencing process, the terminal fragments of the sequence were removed. The 1592 bp region encoding the entire VP3 protein was used for the final analysis. The analysis of the sequencing results was aimed at examining the VP3 protein sequence of GPV strains, which exhibited a similar sequence structure in the conserved region to the sequences of GPV strains circulating in European countries and worldwide. Nucleotide sequences encoding the VP3 capsid protein from 13 GPV strains identified in the examined samples were analyzed. This allowed for the visualization, analysis, and documentation of differences between individual nucleotide sequences. Comparisons of the obtained nucleotide sequences and the numbering of nucleotide and amino acid positions were made for the sequence numbering of the reference strain from Hungary (GenBank Accession No.: NC_001701) and the strain from Germany from 2016 (GenBank Accession No.: KU_684472). Based on the obtained nucleotide sequences of the VP3 gene, a phylogenetic tree was created using the NJ (Neighbor-Joining) method, illustrating the similarity between the sequences of Polish goose parvovirus strains originating from transport vehicles in Poland and the sequences of GPV strains circulating worldwide, included in the GenBank (NCBI) database. The analyzed sequences of GPV strains originating from goose flocks formed four separate groups on the phylogenetic tree, designated as VP3.1–VP3.4 (Figure 3). The VP3.1 group included sequences from strains originating in China. The most numerous group, VP3.2, included sequences from China, Taiwan, Russia, Germany, Hungary (including the reference strain GenBank Accession No.: NC_001701), and nine of the examined GPV sequences. The VP3.3 group consisted of four Chinese strains. The VP3.4 group included the four examined strains, as well as strains from China, Taiwan, and Poland.

A nucleotide similarity of 94.53–99.87% was demonstrated between the examined GPV strain sequences and the sequences from the GenBank (NCBI) database. The nucleotide homology of the VP3 capsid protein gene of all Polish strains to the reference strain (GenBank Accession No: NC_001701) was 94.67–99.94%. However, the nucleotide sequence identity between examined strains GPV and the strains belonging to Asia was 88.66% to 99.56%.

In the next stage of the study, amino acid sequence analysis was performed. The 1592 bp nucleotide chain corresponds to the 350-amino acid sequence of the VP3 protein. The numbering of amino acid positions in the protein chain was referenced to the VP3 sequence of the reference strain (GenBank Accession No.: NC_001701 Amino acids in the protein chain form the structure of proteins, and substituting one amino acid in the amino acid sequence for another amino acid can alter the protein’s function or render the protein nonfunctional by altering its three-dimensional structure. This likely affects the mechanisms of viral pathogenicity. The highest amino acid variability was found in strains 139/20 and 143/20. However, the replacement of T (threonine) with alanine (A) at amino acid position 311 and R (arginine) with tryptophan (W) at position 347 was considered characteristic for the examined strains.

**Figure 4:**
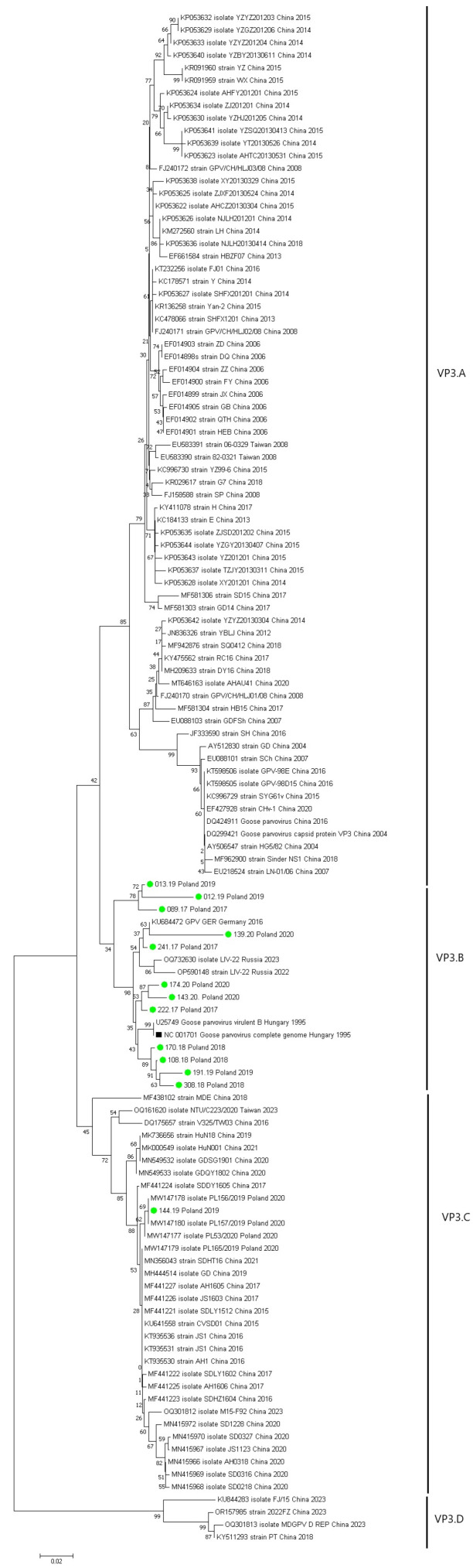
A phylogenetic tree developed based on the amino acid sequence of the VP3 gene of the capsid protein of Polish and foreign GPV strain sequences and the reference strain (marked with a black square). The examined strains are marked with a green circle. Summary diagram (VP1.A–VP1.D).

Based on the obtained nucleotide sequences of the VP3 gene, a phylogenetic tree was created using the NJ (Neighbor-Joining) method, illustrating the similarity between the sequences of Polish goose parvovirus strains originating from transport vehicles in Poland and the sequences of GPV strains circulating worldwide, included in the GenBank (NCBI) database. The analyzed GPV strain sequences formed four separate groups on the phylogenetic tree, designated as VP1.A–VP1.D (Figure 4).

The VP3.B group included 12 examined sequences, a reference sequence from Hungary, sequences from Russia, and a sequence from Germany. The VP3.C group included one examined sequence (144/19) and sequences from China, Taiwan, and Poland. The tree shows that the 12 examined sequences are characterized by high homology to European strains. The amino acid similarity between the Polish GPV isolates was 96.62–99.87% while the homology to the reference strain (NC_001701) was 97.72–99.91%.

**Figure 5:**
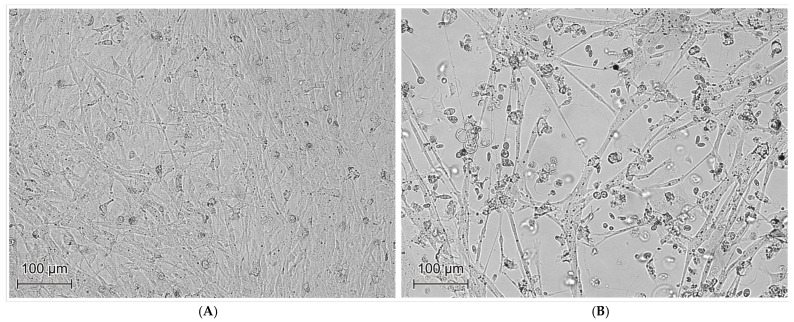
(**A**) Uninfected goose embryo kidney (GEK) cell culture—72 h of incubation (inverted field light microscopy image, magnification 200×). (**B**) Cytopathic effect (CPE) produced by the 222/17 field strain in the GEF culture on the 72nd day of incubation at passage III (inverted field light microscope image, magnification 200×).

**Isolation of GPV strains in goose embryo fibroblast (GEF) cell culture**. After PCR identification, 13 goose parvovirus materials were adapted to goose embryo fibroblast cell cultures. GEF cell cultures showing 80–100% bottle surface coverage (monolayer) were infected with selected virological materials. To adapt GPV to GEFs cultures, a zero passage and three subsequent passages were performed at 72–96 h intervals. The earliest appearance of the cytopathic effect was noted in the first passage at 36–48 h p.i. in GEFs cultures infected with strains: 222/17, 241/17, 308/18, 144/19, and 191/19 (Figure 5). However, after the third passage, a cytopathic effect was observed in the case of strains: 222/17, 241/17, 108/18, 170/18, 308/18, 144/19, 191/19, 143/20, 174/20. No cytopathic effect was observed in the case of strains: 089/17, 012/19, 013/19, 139/20. Control cultures, uninfected with GEF, are shown in (Figure 5B) In the uniform cell layer, changes in cell morphology were observed, including rounding, changes in light refraction (increased cell transparency), cell aggregation, and signs of cell fusion (formation of multinucleated cell syncytia), as well as decreased fibroblast proliferation and increased apoptosis compared to cells uninfected with geese parvovirus. After 96–120 h p.i., the cell layer was destroyed. In our PCR studies, after the third passage, ten positive samples were found (222/17, 241/17, 170/18, 108/18, 308/18, 144/19, 191/19, 143/20, 174/20). After the third passage, no cytopathic effect was noted, and no genetic material was detected by PCR in samples 089/17, 012/19, 013/19, 139/20, which allows us to assume that the collected material was not capable of multiplying in cell cultures. The results are summarized in Table 2 and Table 3. 

**Determination of TCID_50_ infectious titer in GEFs cultures.** The third passage of the examined strains and the reference strain were used to determine the infectious titer. Their titers ranged from 10^2.5^ TCID_50_/0.1 mL to 10^6.8^ TCID_50_/0.1 mL. The titer of the reference strain was 10^4^ TCID_50_/0.1 mL.

## 4. Discussion

Since 1956, Goose Parvovirus (GPV) has been extensively disseminated across Europe, the Americas, and Asia. It exhibits high pathogenicity and lethality in geese and ducks, leading to significant economic losses within the poultry industry [4,14,16,17]. Despite its importance, there is a lack of research on the presence of GPV in the environment, especially in transport vehicles, which may be a source of infection for transported birds, as well as for the environment and wild birds. This study attempted to assess the presence of GPV genetic material, identify strains through molecular analysis, and culture the obtained strains in goose embryo fibroblast cell cultures (GEFs) to assess the potential pathogenicity of strains obtained from transport vehicles. The detection of GPV genetic material and its sequences in the environment of transport trucks was intended to assist in the assessment of transport vehicles as a potential source and reservoir of GPV infections for waterfowl. No such studies have been conducted to date.

During this molecular epidemiological study of Derzsy’s disease, performed for the first time in waterfowl transport vehicles, we found that all isolated strains were goose parvoviruses, which could be considered as strains responsible for causing infection in geese and the occurrence of disease symptoms.

Based on the results obtained in the multiplication of GPV in goose embryo fibroblast (GEFs) cell cultures. GPV propagation using GEF cell cultures is useful, although time-consuming, in virological diagnosis. In the present study, GPV were propagated in cell culture in order to confirm the results obtained by PCR. As a non-enveloped virus, parvovirus releases viral replicates through cell lysis during late infection [18]. The cytotoxic effect is induced primarily by NS1 [5,6,18,19,20,21,22,23,24,25], which plays an important role in the replication process of parvovirus [10]. Changes in infected GEF cell cultures demonstrated effective replication of the virus.

The results of the study conducted by Tatár-kis et al. in 2004 [26] show that Hungarian strains are characterized by a low degree of evolution, relatively low differentiation, and high homology to each other. This is reflected in our results, as the results of phylogenetic analysis and epidemiological data (usually a lack of vaccination in selected years due to difficult access to commercial GPV vaccines) exclude the possibility of resolution of the vaccine virus. The nucleotide similarity between the sequence of strain B isolated in the 1960s and the reference strain (NC_001701) and the sequences of our isolates is 94.67–99.94%. This suggests that contemporary Hungarian and Polish GPV strains shared a common ancestor, and that the DNA sequences of different GPV isolates are highly conserved. Despite the low level of genetic variability, well-defined genogroups could be identified in the phylogenetic trees. Based on the nucleotide sequences of the VP3 protein gene, the Polish strains obtained were part of the VP3.3 group and were grouped together on a single branch, along with strains from Hungary, Russia, and Germany. Strains 089/17, 012/19, and 013/19, on the other hand, belong to the VP3.4 group and cluster closely together among the Asian strains. Interestingly, strain 144/19 was the only one that showed a close relationship to the compared strains from Poland. As expected, the Hungarian and Polish strains are characterized by a very low rate of evolution, with no measurable changes in antigenicity or virulence.

The primary role of transport vehicles (Figure 1) that transport birds between the hatchery and the farm, as well as between the farm and the slaughter plant, is to ensure high biosecurity standards for both the birds being transported and the surrounding environment. Waterfowl transport biosecurity involves strict cleaning and disinfection of equipment and vehicles, route planning to avoid high-risk areas, and minimizing contact with other birds to prevent the spread of the disease. Transport elements must be cleaned and disinfected before birds are placed in them. This will minimize the risk of waterfowl being exposed to a contaminated environment. It is extremely important to choose the right cleaning and disinfection techniques to avoid the risk of contaminating the cleaned equipment. Studies conducted by Dzieciolowski et al. [20] have shown that one of the best ways to reduce potential microbial contamination of poultry transport crates is to use hot air, and sodium hypochloride and/or peracetic acid. The decontamination process should also be carried out throughout the entire transport vehicle, which will significantly reduce the risk of pathogens being transmitted by equipment. In addition, people involved in the handling and transport of waterfowl should follow recommendations and measures concerning personal hygiene [11,27]. Waterfowl loaded onto transport vehicles usually show no clinical signs, which suggests that they are healthy. Therefore, the key task of proper disinfection of transport vehicles is to prevent the presence of pathogens resistant to environmental conditions, such as goose parvovirus. At the same time, preliminary scientific studies conducted by EFSA indicated that transporting birds in the confined space of a transport vehicle for more than 8 h significantly increases the risk of potential infection with pathogens [23]. The potential risk of infection in birds may depend on several factors, other than proper and effective disinfection. These factors include: the number of birds in the cage and/or transport vehicle; proper securing of the transport vehicle while in transit; adequate ventilation inside the transport cabin; and climatic conditions. Vehicles used to transport poultry are not free from contamination, as they are surrounded by an environment full of contaminants that are transferred from the air to the surface on which they travel, increasing the risk of contamination. Therefore, microbiological control in vehicles must focus on necessary and effective measures to break the chain of transmission, especially of pathogenic or opportunistic microorganisms, as well as potentially infectious ones. To meet this need, technologies and strategies are available to ensure the adequate microbiological quality of vehicles used to transport poultry, including waterfowl viral pathogens [4,14,16,17,27,28,29]. At the same time, it should be noted that the success of antimicrobial protection is not limited to proper cleaning and disinfection of transport vehicles and proper personal hygiene of transport personnel. Optimizing biometeorological parameters is also crucial, with the most important being the appropriate ambient temperature, which ensures maximum comfort for poultry during transport. In many countries, especially those with warm climate conditions, poultry has been transported in open vehicles without control of meteorological variables, which can potentially lead to an increase in the internal temperature of vehicle cargo during transport [21,22]. Studies conducted by Hirakawa et al. have shown that heat stress in poultry significantly weakens the immune system, causing damage to lymphoid tissues (thymus, bursa of Fabricius), which are crucial for the development of B and T lymphocytes. This damage leads to the physical atrophy of these tissues, lymphocyte depletion, and impairment of the cells’ ability to mature, proliferate, and differentiate into functional immune cells. As a result, birds exhibit a weakened immune response, including reduced antibody production, including post-vaccination antibodies [28]. Such studies have not yet been conducted on waterfowl species; however, it can be assumed that the effects of heat stress will be similar, as ducks and geese are significantly more sensitive to potential overheating. Therefore, contamination of transport vehicles and poultry cages with pathogens carries the potential for a high risk of infection during bird movement.

## 5. Conclusions

This study identified the presence of GPV genetic material in vehicles transporting waterfowl and initially determined its pathogenicity by culturing the material in goose embryo fibroblast (GEF) cell cultures. This demonstrated that transport components are significant vectors of pathogens, particularly for goose parvovirus. Preliminary studies on the presence of GPV genetic material in transport vehicles suggest the need to implement effective microbiological surveillance procedures during the preparation, handling, and transport of poultry from the hatchery to the farm and from the farm to the slaughterhouse. This will ensure adequate protection of birds from potential infection. Therefore, effective biosecurity measures during poultry transport are crucial to prevent the spread of viral infections.

## Figures and Tables

**Table 1:** Summary of the number of vehicles examined in 2017–2020, the percentage distribution of positive samples in a given year compared to the samples collected in a given year and the percentage of samples subjected to sequence analysis.

Year	Number of Vehicles Examined	Number of Vehicles Testing Positive for GPV	Number of Sequences Obtained	Accession Number GenBANK
2017	15	6 (40%)	3 (50%)	waiting
2018	22	9 (40.91%)	3 (33.34)	waiting
2019	17	5 (29.41%)	4 (80%)	waiting
2020	12	5 (41.67%)	3 (60%)	waiting
**Total**	**66**	**25 (37.87%)**	**13**	

**Table 2:** The amino acid mutations of different GPV strains isolated in Poland between 2017 and 2020.

Lp.	Amino Acid Position	Amino Acid of Reference Strain Sequence *	Amino Acid of Field Strain	Strains with Mutation Detected
1	20	G	S	139.20_Poland_2020
2	21	N	H	139.20_Poland_2020
3	22	A	G	139.20_Poland_2020
4	25	N	T	139.20_Poland_2020
5	26	W	S	308.18_Poland_2018
6	29	D	E	139.20_Poland_2020
7	30	S	N	139.20_Poland_2020
8	31	Q	V	139.20_Poland_2020
9	56	K	Q	139.20_Poland_2020
10	58	I	L	143.20_Poland_2020
11	59	T	P	143.20_Poland_2020
12	60	S	R	174.20_Poland_2020 012.19_Poland_2019
			G	143.20_Poland_2020
13	61	G	A	143.20_Poland_2020
14	64	Q	D	012.19_Poland_2019
15	65	D	H	012.19_Poland_2019
16	67	N	Y	143.20_Poland_2020
17	70	Y	F	143.20_Poland_2020
18	77	W	G	012.19_Poland_2019
19	80	F	L	012.19_Poland_2019
20	83	N	C	012.19_Poland_2019
21	94	W	G	139.20_Poland_2020
22	95	Q	L	012.19_Poland_2019
23	99	N	S	139.20_Poland_2020
24	103	G	E	012.19_Poland_2019
25	104	I	F	012.19_Poland_2019
26	126	Q	H	139.20_Poland_2020
27	127	T	A	139.20_Poland_2020
28	192	C	R	144.19_Poland_2019 012.19_Poland_2019 013.19_Poland_2019 089.17_Poland_2017
29	204	A	V	191.19_Poland_2019 108.18_Poland_2018 170.18_Poland_2018 308.18_Poland_2018
30	205	K	E	144.19_Poland_2019 012.19_Poland_2019 013.19_Poland_2019 089.17_Poland_2017
31	208	Q	R	139.20_Poland_2020
32	222	P	S	191.19_Poland_2019 108.18_Poland_2018 170.18_Poland_2018 308.18_Poland_2018
33	231	L	P	139.20_Poland_2020 174.20_Poland_2020 143.20_Poland_2020 241.17_Poland_2017 222.17_Poland_2017
34	232	R	G	012.19_Poland_2019
35	235	D	Y	144.19_Poland_2019 012.19_Poland_2019 013.19_Poland_2019 089.17_Poland_2017
36	236	E	K	139.20_Poland_2020
37	253	Q	H	139.20_Poland_2020
38	260	G	S	144.19_Poland_2019 012.19_Poland_2019 013.19_Poland_2019 089.17_Poland_2017
39	261	C	R	139.20_Poland_2020 174.20_Poland_2020 143.20_Poland_2020 241.17_Poland_2017 222.17_Poland_2017
40	262	E	K	308.18_Poland_2018
41	266	W	C	139.20_Poland_2020
42	270	P	S	144.19_Poland_2019 013.19_Poland_2019
43	275	R	G	144.19_Poland_2019 012.19_Poland_2019 013.19_Poland_2019 089.17_Poland_2017
44	282	K	E	144.19_Poland_2019 012.19_Poland_2019 013.19_Poland_2019 089.17_Poland_2017
45	293	L	Q	139.20_Poland_2020 174.20_Poland_2020 143.20_Poland_2020
			P	191.19_Poland_2019 108.18_Poland_2018 170.18_Poland_2018 308.18_Poland_2018
46	308	E	K	139.20_Poland_2020 174.20_Poland_2020 143.20_Poland_2020 144.19_Poland_2019 012.19_Poland_2019 013.19_Poland_2019 089.17_Poland_2017 241.17_Poland_2017 222.17_Poland_2017
47	309	R	G	144.19_Poland_2019 012.19_Poland_2019 013.19_Poland_2019 089.17_Poland_2017
48	311	T	A	all sequences
49	323	L	S	139.20_Poland_2020 174.20_Poland_2020 143.20_Poland_2020 144.19_Poland_2019 012.19_Poland_2019 013.19_Poland_2019 089.17_Poland_2017 241.17_Poland_2017 222.17_Poland_2017
50	326	R	M	012.19_Poland_2019
51	332	S	P	144.19_Poland_2019 012.19_Poland_2019 013.19_Poland_2019 089.17_Poland_2017
52	333	S	G	139.20_Poland_2020 174.20_Poland_2020 143.20_Poland_2020 222.17_Poland_2017
53	335	K	E	144.19_Poland_2019 012.19_Poland_2019 013.19_Poland_2019 089.17_Poland_2017
54	347	R	W	all sequences
55	351	S	R	139.20_Poland_2020
56	352	R	W	191.19_Poland_2019 108.18_Poland_2018 170.18_Poland_2018 308.18_Poland_2018
57	356	H	Y	144.19_Poland_2019 012.19_Poland_2019 013.19_Poland_2019 089.17_Poland_2017
			D	222.17_Poland_2017
58	358	G	V	012.19_Poland_2019
59	361	K	T	all sequences except 222.17_Poland_2017
60	362	N	S	144.19_Poland_2019 012.19_Poland_2019 013.19_Poland_2019 089.17_Poland_2017
61	363	K	R	139.20_Poland_2020 144.19_Poland_2019 013.19_Poland_2019
62	367	L	P	144.19_Poland_2019 012.19_Poland_2019 013.19_Poland_2019 089.17_Poland_2017
63	368	Q	R	144.19_Poland_2019 012.19_Poland_2019 013.19_Poland_2019 089.17_Poland_2017
64	370	E	Q	191.19_Poland_2019 108.18_Poland_2018 170.18_Poland_2018 308.18_Poland_2018
65	382	M	I	139.20_Poland_2020
66	396	M	K	139.20_Poland_2020 241.17_Poland_2017
			R	144.19_Poland_2019
67	397	F	S	191.19_Poland_2019 308.18_Poland_2018
68	405	F	C	144.19_Poland_2019
69	408	T	A	241.17_Poland_2017
70	409	G	E	144.19_Poland_2019
71	426	V	A	139.20_Poland_2020 191.19_Poland_2019 108.18_Poland_2018 308.18_Poland_2018
72	432	R	Q	144.19_Poland_2019
73	441	I	Q	174.20_Poland_2020
74	442	H	N	174.20_Poland_2020
75	444	R	H	144.19_Poland_2019 089.17_Poland_2017
76	446	C	S	144.19_Poland_2019
77	453	C	Y	144.19_Poland_2019 089.17_Poland_2017
78	461	T	M	144.19_Poland_2019
79	467	I	T	144.19_Poland_2019
80	471	P	L	139.20_Poland_2020 144.19_Poland_2019
81	472	S	N	144.19_Poland_2019
82	475	R	Q	144.19_Poland_2019
83	481	R	K	144.19_Poland_2019
84	508	Q	L	144.19_Poland_2019 089.17_Poland_2017
85	509	A	V	144.19_Poland_2019 089.17_Poland_2017
86	512	L	K	191.19_Poland_2019
87	518	V	L	191.19_Poland_2019
88	523	I	T	191.19_Poland_2019
89	529	D	A	191.19_Poland_2019
90	530	I	R	191.19_Poland_2019
91	535	C	R	191.19_Poland_2019
			L	308.18_Poland_2018

The legend: * the strain from Hungary (Acc. No. U25749) was used as the reference strain; A—Alanine; R—Arginine; N—Asparagine; D—Aspartic acid; C—Cysteine; E—Glutamic acid; Q—Glutamine; G—Glycine; H—Histidine; I—Isoleucine; L—Leucine; K—Lysine; M—Methionine; F—Phenylalanine; P—Proline; S—Serine; T—Threonine; W—Tryptophan; Y—Tyrosine; V—Valine.

**Table 3:** Virus isolation in goose embryo fibroblast (GEF) cell culture.

Lp.	Inoculum	Cytopathic Effect (CPE) at First Passage	Cytopathic Effect (CPE) at Third Passage	Presence of GPV Genetic Material After the Third Passage
1	089/17	negative	negative	negative
2	222/17	positive	positive	positive
3	241/17	positive	positive	positive
4	108/18	negative	positive	positive
5	170/18	negative	positive	positive
6	308/18	positive	positive	positive
7	012/19	negative	negative	negative
8	013/19	negative	negative	negative
9	144/19	positive	positive	positive
10	191/19	positive	positive	positive
11	139/20	negative	negative	negative
12	143/20	negative	positive	positive
13	174/20	negative	positive	positive

## Data Availability

The original contributions presented in this study are included in the article. Further inquiries can be directed to the corresponding author.
